# Deficiency of Nucleotide-binding oligomerization domain-containing proteins (NOD) 1 and 2 reduces atherosclerosis

**DOI:** 10.1007/s00395-020-0806-2

**Published:** 2020-06-25

**Authors:** Ann-Kathrin Vlacil, Jutta Schuett, Volker Ruppert, Muhidien Soufi, Raghav Oberoi, Kinan Shahin, Christian Wächter, Thomas Tschernig, Yu Lei, Fan Liu, Uwe J. F. Tietge, Bernhard Schieffer, Harald Schuett, Karsten Grote

**Affiliations:** 1 Cardiology and Angiology, Philipps-University Marburg, Hans-Meerwein-Straße 2, 35043 Marburg, Germany; 2 Faculty of Medicine, Institute for Anatomy and Cell Biology, Saarland University, Campus Homburg/Saar, Saarbrücken, Germany; 3 Division of Clinical Chemistry, Department of Laboratory Medicine, Karolinska Institutet, Stockholm, Sweden; 4 Clinical Chemistry, Karolinska University Laboratory, Karolinska University Hospital, Stockholm, Sweden

**Keywords:** Atherosclerosis, Immune system, Macrophage, Foam cells, Cholesterol synthesis/absorption, Cardiovascular diseases

## Abstract

Atherosclerosis is crucially fueled by inflammatory pathways including pattern recognition receptor (PRR)-related signaling of the innate immune system. Currently, the impact of the cytoplasmic PRRs nucleotide-binding oligomerization domain-containing protein (NOD) 1 and 2 is incompletely characterized. We, therefore, generated *Nod1*/*Nod2* double knockout mice on a low-density lipoprotein receptor (*Ldlr*)-deficient background (= *Ldlr*^−/−^*Nod1/2*^−/−^) which were subsequently analyzed regarding experimental atherosclerosis, lipid metabolism, insulin resistance and gut microbiota composition. Compared to *Ldlr*^−/−^ mice, *Ldlr*^−/−^*Nod1/2*^−/−^ mice showed reduced plasma lipids and increased hepatic expression of the scavenger receptor LDL receptor-related protein 1 after feeding a high-fat diet for 12 weeks. Furthermore, intestinal cholesterol and its bacterial degradation product coprostanol were elevated in *Ldlr*^−/−^*Nod1/2*^−/−^ mice, correlating with the increased abundance of *Eubacterium coprostanoligenes* as assessed by 3rd generation sequencing of the gut microbiota. Atherosclerotic plaques of *Ldlr*^−/−^*Nod1/2*^−/−^ mice exhibited less lipid deposition and macrophage accumulation. Moreover, macrophages from *Ldlr*^−/−^*Nod1/2*^−/−^ mice showed higher expression of the cholesterol efflux transporters *Abca1* and *Abcg1* and accordingly reduced foam cell formation. Deficiency of *Nod1* and *Nod2* led to reduced plaque lipid deposition and inflammatory cell infiltration in atherosclerotic plaques. This might be explained by diminished plasma lipid levels and foam cell formation due to altered expression of key regulators of the hepatic cholesterol pathway as well as differential intestinal cholesterol metabolism and microbiota composition.

**Electronic supplementary material:**

The online version of this article (10.1007/s00395-020-0806-2) contains supplementary material, which is available to authorized users.

## Introduction

Elevated levels of blood cholesterol and vascular inflammation are considered as the primary triggers of cardiovascular disease due to atherosclerosis, a chronic disease of arteries [15]. Subsequent fatal events such as myocardial infarction are still responsible for the most illness-related fatalities worldwide [28]. There is an ongoing debate on the weighting of trigger mechanisms or whether they equally contribute to the initiation and progression of atherosclerosis. In this regard, cholesterol-lowering strategies, such as statins, are widely used as primary as well as secondary prevention in patients with imminent or apparent atherosclerotic cardiovascular disease since the 90s of the past century [1]. Various pleiotropic effects—including anti-inflammatory effects—of statins have been reported; however, cholesterol-lowering is regarded as their principle mechanism of action. Although the inflammatory nature of atherosclerosis is known for quite some time, the first successful anti-inflammatory therapy for atherosclerotic disease using a monoclonal antibody targeting interleukin (IL)-1β has been reported just in 2017 [27].

Nucleotide-binding oligomerization domain-containing protein (NOD) are pattern-recognition receptors (PRR), and NOD1 and NOD2, previously known as caspase activation and recruitment (CARD) domain family member CARD4 and CARD15, represent the founding members of the NOD-like receptor family. Different to other membrane bound PRRs, they operate as soluble cytosolic receptors recognizing conserved bacterial peptidoglycan fragments (muropeptides) in order to initiate an adequate immune response [8, 25]. Expression of *Nod1* and *Nod2* has not only been shown in immune cells such as macrophages but also in vascular cells such as endothelial cells and smooth muscle cells [19, 30]. Both NOD1 and NOD2 interact with the adapter protein receptor-interacting serine/threonine-protein kinase 2 and subsequently share a common downstream signaling cascade involving nuclear factor (NF)-κB and mitogen-activated protein kinase (MAPK) signaling pathways [12]. Important roles for NOD1 and NOD2 in various experimental disease models have been identified in recent years including their involvement in the regulation of the intestinal barrier function and the gut microbiota composition [22].

The role of *Nod1* and *Nod2* in atherosclerosis and insulin resistance has already been investigated in several experimental studies; however, with partially conflicting results. Administration of a NOD1 agonist enhanced atherosclerosis in apolipoprotein E (*Apoe*)-deficient mice [17], whereas *Nod1/Apoe-*deficient mice showed reduced atherosclerosis [13, 17]. Following antibiotic pretreatment, NOD2 stimulation in addition to an oral gavage with the periodontal pathogen *Porphyromonas gingivalis* decreased atherosclerosis in Apoe-deficient mice, whereas *P. gingivalis* alone increased atherosclerosis in *Nod2*/*Apoe*-deficient mice [32]. Contrarily, Johansson et al. showed that stimulation with a NOD2 ligand aggravated atherosclerosis in low-density lipoprotein receptor (*Ldlr*)-deficient mice [16]. In regard to insulin resistance, Schertzer et al. [29] reported that especially NOD1 ligands induce insulin resistance, whereas NOD1/2-deficient mice were protected from high-fat diet-induced insulin resistance. Likewise, high-fat diet (HFD)-induced translocation of intestinal bacteria into adipose tissue and blood as well as insulin resistance is prevented in NOD1-deficient mice [3]. Opposing results were seen by Cavallari et al. [9] NOD2 stimulation reduced insulin resistance, whereas NOD1 stimulation worsened insulin resistance in obese mice without altering the composition of the microbiome.

The partially contradicting outcomes of these studies can potentially be attributed to different experimental settings, e.g. for experimental atherosclerosis different knockout (KO) animal models (*Apoe*-KO, *Ldlr*-KO mice, systemic vs. bone marrow-specific KO), with or without antibiotic pretreatment and with or without different NOD ligand or bacterial pathogen administration regimes were used. Besides periodontal pathogens, the gut microbiota have been shown to contribute to atherosclerosis and type 2 diabetes [21]. However, an exogenously applied bacterial load or NOD ligands in experimental models principally mimic severe systemic infection rather than a low-grade bacterial load. The latter presents a chronically evolving cardiovascular risk and more likely resembles the situation in patients. Most studies with NOD-deficient mice used a NOD-single knock-out even though bacterial-derived peptidoglycan motifs mostly activate both NOD receptors. Although NOD1 and NOD2 may be activated by different ligands, compensatory effects are conceivable due to joint down-stream signaling. Hence, in the present study we investigated hypercholesterolemic mice deficient for *Nod1* as well as for *Nod2* in regard to experimental atherosclerosis, lipid metabolism, insulin resistance and gut microbiota composition.

## Methods

### Reagents

The NOD-ligands, l-Ala-γ-D-Glu-meso-diaminopimelic acid (TriDAP, NOD1) and muramyl dipeptide (MDP, NOD2), were purchased from Invivogen (San Diego, CA).

### Mice and cells

Low-density lipoprotein receptor-deficient (*Ldlr*^*−/−*^, B6.129S7-Ldlr^tm1Her^/J) and *Nod1*^−/−^, *Nod2*^−/−^, *Ldlr*^−/−^ = *Ldlr*^−/−^*Nod1/2*^−/−^ (B6.129S7-Ldlr^tm1Her^/J, B6.129P2-Nod1^tm1Inoh^/MakJ, B6.129S1-Nod2^tm1Flv^/J) mice were bred and maintained under pathogen-free conditions in 12 h dark/12 h light cycle in the animal facility at Philipps-University Marburg. All experiments were approved by the governmental animal ethics committee (50/2015) and conform to the guidelines from directive 2010/63/EU of the European Parliament. At the age of 10 weeks, male mice were fed a HFD (D12079B, containing 21% fat and 0.21% cholesterol, Research Diets, New Brunswick, NJ) for 12 weeks.

10-week-old *Ldlr*^*−/−*^ mice were i.v. injected with the NOD-ligands Tri-DAP and MDP (each 100 µg/mouse). Livers for real-time PCR analysis were collected after 6 h.

Bone marrow-derived macrophages (BMDM) were isolated from tibia and femur and 2 × 10^6^ cells were cultured in RPMI (Roswell Park Memorial Institute) 1640-GlutaMAX (Life Technologies™, Darmstadt, Germany), 10% fetal calf serum (FCS, PAN-Biotech, Aidenbach, Germany), 1% penicillin/streptomycin (100 U/mL and 100 mg/mL, Sigma-Aldrich) and 10% supernatant from the fibroblast cell line L929 in 6-well plates. After 6 days, cells were controlled for purity (> 90% CD11b^+^) by flow cytometry and used for further experiments.

### Glucose tolerance and insulin resistance

Glucose tolerance and insulin resistance tests were performed after 6 and 12 weeks of HFD on two consecutive days. Mice were fasted for 4 h and baseline glucose measurement was taken from a drop of tail vein blood using an OneTouch Ultra2 glucometer (LifeScan Inc. Milpitas, CA). Afterwards, mice were injected i.p. with 1.25 g glucose or 0.75 IU of insulin (Sanofi, Frankfurt, Germany) per kg body weight in a total volume of 200 μL buffered saline (PBS). Following injection, blood glucose was measured over a period of 2 h as stated above.

### Plasma lipid analysis

Plasma samples were collected after 4 h of fasting and plasma total cholesterol and triglyceride levels were measured enzymatically using commercially available reagents (Roche Diagnostics, Mannheim, Germany) as reported previously [23]. Plasma samples were subjected to fast protein liquid chromatography gel filtration on an Äkta purifier equipped with a Superose 6 column (GE Healthcare, Little Chalfont, UK). Chromatography was done at a flow rate of 0.5 mL/min and lipoprotein fractions of 500 µL each were collected and assayed for cholesterol concentrations. Individual lipoprotein subclasses were isolated by tabletop sequential ultracentrifugation from 30 µL of plasma using a TLA-120.2 fixed-angle rotor (Beckman Coulter, Woerden, The Netherlands) and a 1.34 g/mL potassium bromide stock solution to adjust densities (densities, VLDL/IDL: 1.006 < *d* < 1.019, LDL: 1.019 < *d* < 1.063, HDL: 1.063 < *d* < 1.21; each run 3 h at 100,000 rpm). After extensive dialysis against PBS, cholesterol concentrations within individual fractions were determined using a commercial colorimetric assay (Roche Diagnostics).

### Fecal sterol analysis

Fecal samples were weighed and aliquots separated into bile acid and neutral sterol fractions as reported previously [10]. Briefly, samples (50 mg each) were first heated for 2 h at 80 °C in 1 mL alkaline methanol with 5*α*-cholestane added as internal standard and then extracted three times with petroleum ether 60–80. The top layer (ether) was used for neutral sterol analysis and the bottom layer (water) to profile bile acid species. The ether phase was dried under a stream of nitrogen, then a mixture of *N*,*O*-bis(trimethylsilyl)trifluoroacetamide, pyridine and trimethylchlorsilan (5:5:0.1) was added followed by drying under nitrogen again; finally, samples were taken up in heptane. The water phase was transferred to Sep-Pak C18 cartridges, flushed out with 75% methanol followed by drying the eluate under a stream of nitrogen, and derivatized as above. Sterol analyses were performed by gas–liquid chromatography (Agilent, Santa Clara, CA) with a Chrompack CPSil19 column, Helium as carrier gas and including calibration curves for all analytes determined (covering a range between 0 and 50 nmol) [10].

### Tissue preparation

After 4 h of starvation, blood samples were collected and subsequently mice were killed and aorta was perfused with PBS after opening of the hepatic portal vein. Heart, including aortic root and aortic arch, was dissected after perfusion with PBS. The aortic arch was separated, snap-frozen in liquid nitrogen and the heart including aortic root was embedded in Tissue Tek OCT (Sakura Finetek, Staufen, Germany) for histochemistry and immunohistochemistry. Organs and tissues including liver, cecum, ileum and aortic arch were collected into cryotubes, flash frozen and stored at − 80 °C.

### Histochemistry and immunohistochemistry

Atherosclerotic burden was quantified in the aortic root by Oil Red O (Sigma-Aldrich) staining. Within the aortic root, serial cryostat sections (8 μm, CM3050S, Leica Microsystems) at the level of all three cusps were prepared and atherosclerotic lesions were analyzed by Oil Red O staining (2 h at 60 °C) for the measurement of atherosclerotic plaque burden. The remaining sections were used for immunohistochemical analysis of the atherosclerotic plaque composition. Air-dried sections were fixed in ice-cold acetone and stained with antibodies against MOMA-2 (reacts with monocytes/macrophages, Acris Antibodies GmbH, Herford, Germany) or α-smooth muscle actin (Abcam, Cambridge, UK), visualized by appropriate secondary antibodies (ImmPRESS™ detection reagent, Vector Laboratories, Burlingame, CA) and counterstained with hematoxylin (solution Gill No. 2, Merck KGaA, Darmstadt, Germany). Sirius Red staining was used to visualize collagen content in atherosclerotic plaques. Briefly, tissue sections were incubated with 0.1% Sirius Red F3BA (Sigma–Aldrich) in saturated picric acid for 1 h and then rinsed with 0.01 N HCl for 1 min twice. The sections were then dehydrated with 70% ethanol for 30 s. Structural mature type I collagen appeared bright orange-red in polarized light. Morphometric data on three sections were obtained using a light microscope (DMI3000 B and DM4000 B microscope, Leica Microsystems Wetzlar, Germany) and ImageJ software (National Institutes of Health, Bethesda, MD). The atherosclerotic plaque size was determined by calculating the percentage of the Oil Red O positive area of the total aortic root cross sectional area. MOMA-2, α-smooth muscle cell actin and Sirius Red were expressed as percentage of total plaque area.

### Real-time PCR

Total RNA from snap-frozen liver, ileum or bone marrow-

derived macrophages was isolated using RNA-Solv^®^ Reagent (Omega Bio-tek, Norcross, GA) following the manufacturer’s instructions and reverse-transcribed with SuperScript reverse transcriptase, oligo(dT) primers (Thermo Fisher Scientific, Waltham, MA) and deoxynucleoside triphosphates (Promega, Mannheim, Germany). Real-time PCR was performed in duplicates in a total volume of 20 µL using Power SYBR green PCR master mixture (Thermo Fisher Scientific) or TaqMan Fast Advanced Master Mix (Applied Biosystems, Foster City, CA) on a Step One Plus Real-time PCR system (Applied Biosystems) in 96-well PCR plates (Applied Biosystems). Real-time PCR using SYBR green was performed with an initial denaturation step at 95 °C for 10 min, followed by 40 PCR cycles that consisted of 95 °C for 15 s, 60 °C for 1 min and 72 °C for 10 s. In terms of TaqMan Fast Advanced Master Mix, real-time PCR was performed with an initial holding and denaturation step at 50 °C for 2 min followed by 20 s at 95 °C. The 40 PCR cycles consisted of 95 °C for 1 s and 60 °C for 20 s. SYBR Green and Taq emissions were monitored after each cycle. For normalization, expression of GAPDH was determined in duplicates. Relative gene expression was calculated using the $2^{{ - \Delta \Delta C_{{\text{t}}} }}$ method. Real-time PCR primers were obtained from Microsynth AG (Balgach, Switzerland) and sequences are available in the Online supplementary methods.

### Foam cell formation assay and measurement of cholesterol efflux

Confluent BMDM in 6-well plates were incubated for 4 h in RPMI 1640-GlutaMAX with 1% FCS and 10 µg/mL 1,1ʹ-dioctadecyl-3,3,3ʹ,3ʹ-tetramethylindocarbo-cyanine perchlorate (dil)-labeled human oxLDL (IntracelResources, Frederick, MD) at 37 °C and 5% CO_2_. Afterwards, cells were washed three times with medium and 10 high-power field images per well were taken using an inverted microscope (AxioObserver.Z1, Carl Zeiss, Microimaging, Jena, Germany) and a digital camera (AxioCamMRm, Carl Zeiss). Dil-positive cells were counted using ImageJ software. For flow cytometry analysis, cells were detached using 0.05% Trypsin-ethylenediaminetetraacetic acid (EDTA) and washed with PBS containing 2% FCS. Purity of BMDM was determined by flow cytometry as stated above. Dil-positive cells were assessed using PE fluorescence channel. Cells were acquired on a FACS LSRII flow cytometer (BD Biosciences, San Jose, CA) and analyzed with FlowJo software (Tree Star Inc., Ashland, OR).

Cholesterol efflux was determined as published previously [4]. Experiments were performed in triplicates using BMDM. BMDM were plated in 48-well plates (Corning, Corning, NY) and, after reaching confluence, loaded with ^3^H-cholesterol (1 μCi/mL, NEN Life Sciences Products, Boston, MA) as well as acetylated LDL (50 µg protein/mL) for 24 h to generate foam cells. After washing with PBS, cells were equilibrated in RPMI with 0.2% BSA for 18 h. After another washing with PBS, HDL (50 µg protein/mL, isolated from pooled healthy donors by ultracentrifugation) was added as acceptor. After 5 h, radioactivity within the supernatant was determined by liquid scintillation counting (Beckman LS6500, Beckman Instruments, Palo Alto, CA). Next, 0.1 M NaOH was added to the wells, plates were incubated for 30 min at room temperature and the radioactivity remaining within the cells was assessed by liquid scintillation counting. Efflux is expressed as the percentage of counts recovered from the medium in relation to the total counts present on the plate (sum of medium and cells). Values for unspecific efflux determined as release of ^3^H-cholesterol from macrophages in the absence of HDL were subtracted from the individual values.

### Microbiome analysis

From each mouse, 200 mg cecum was used and DNA was isolated with Stool-lysing Buffer (ASL) (QIAGEN) extracted with Phenol/Chlorophorm/Isomamylalcohol (Roth) and cleaned up with AMPure XP beads (Beckman Coulter, Krefeld, Germany). After concentration measurement and quality control, 16S rRNA—PCR amplification with Nanopore Barcode-Primer was performed (LSK-RAB204) (Oxford Nanopore, Oxford, UK). Afterwards, amplification products were controlled using gel electrophoresis. For sequencing, the sequencing library with a total amount of 100 fmoles PCR ampliconsDNA was prepared according to the instructions of the manufacturer and loaded onto a MinION FlowCell (Oxford Nanopore). In total, 1 × 10^6^ sequences per sequencing run were detected. For basecalling Oxford Nanopore Technology’s official command-line Albacore enabling FAST5 to FASTQ conversion was used. Afterwards FASTQ files were compiled and converted into FASTA files which were used for comparison with the local blast 16S rRNA database. Analysis was performed using MEGAN6 microbiome analysis tool from Eberhard Karls Universität Tübingen.

### Statistical analysis

All data are represented as means ± SD. After normal distribution validation, groups were compared using parametric 2-tailed Student *t*-test or nonparametric Mann Whitney test as appropriate (GraphPad Prism, version 6.05; GraphPad Software, La Jolla, CA). A value of *P* < 0.05 was considered statistically significant. Real-time PCR was performed in technical duplicates.

## Results

### *Nod1/2*-deficiency lowers plasma cholesterol levels

**Fig. 1:**
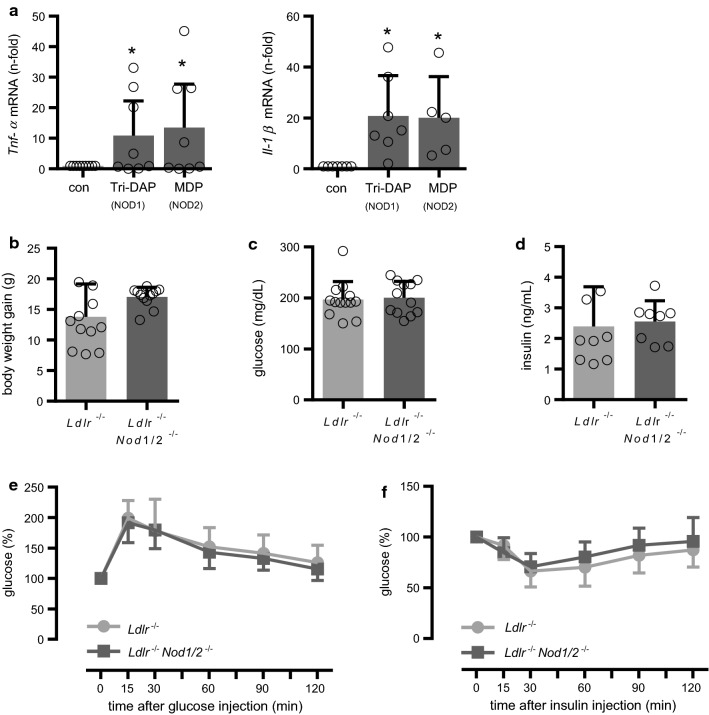
NOD-induced gene expression in BMDM and no effects of Nod1/2-deficiency on body weight and glucose metabolism. **a** *Tnf-α* and *Il-1β* mRNA levels in BMDM from C57BL/6 were stimulated with the NOD1 agonist Tri-DAP or the NOD2 agonist MDP (each 10 µg/mL) and analyzed by real-time PCR, con = control. *Ldlr*^−/−^ and *Ldlr*^−/−^*Nod1/2*^−/−^ mice were fed a HFD for 12 weeks. **b** Body weight gain **c** fasting glucose levels and **d** fasting insulin levels. Plasma glucose levels measured for **e** glucose tolerance after glucose injection (1.25 mg/kg body weight, i.p.) and **f** insulin resistance after insulin injection (0.75 IU/kg body weight, i.p.) at the indicated times. Data were analyzed by Student *t*-test or Mann Whitney test, *P < 0.05

**Fig. 2:**
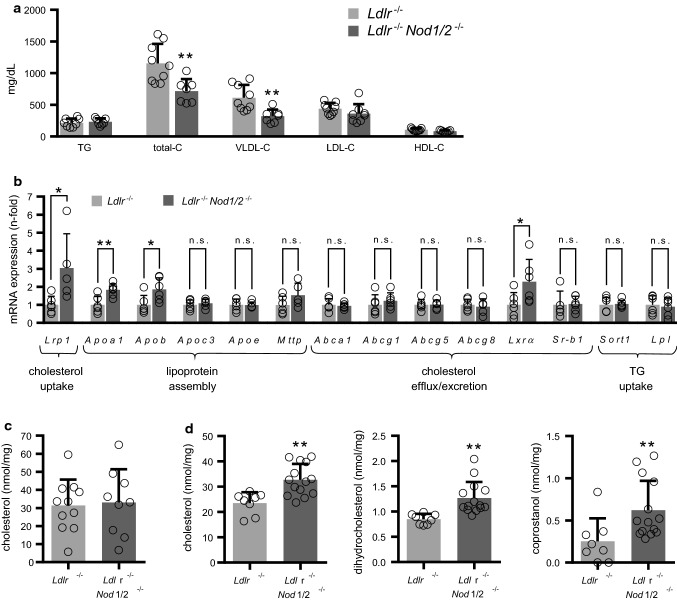
Nod1/2-deficiency lowers plasma cholesterol and increases intestinal cholesterol levels. *Ldlr*^−/−^ and *Ldlr*^−/−^*Nod1/2*^−/−^ mice were fed a HFD for 12 weeks. **a** Lipid levels from plasma samples were analyzed by sequential ultracentrifugation. *TG *triglycerides, *total-C *total cholesterol, *VLDL-C *very-low-density lipoprotein cholesterol, *LDL-C *low-density lipoprotein cholesterol, *HDL-C *high-density lipoprotein cholesterol. **b** Expression levels of key factors for cholesterol and triglyceride metabolism in livers were analyzed by real-time PCR, **c** Cholesterol levels from liver samples were analyzed by sequential ultracentrifugation. **d** Cholesterol and cholesterol derivate levels from feces samples from cecum were analyzed by gas–liquid chromatography. Data were analyzed by Student *t*-test or Mann Whitney test, *P < 0.05, **P < 0.01

NOD1- and NOD2-signaling share many similarities in the initiation of inflammatory pathways [12, 24]. Accordingly, we observed a comparable induction of inflammatory genes in bone marrow-derived macrophages (BMDM) such as tumor necrosis factor-α (*Tnf-α*) and interleukin-1β (*Il-1β*) by the NOD1 ligand l-Ala-γ-d-Glu-meso-diaminopimelic acid (Tri-DAP) and the NOD2 ligand muramyl dipeptide (MDP, Fig. 1a). Previous studies with *Nod1-* or *Nod2*-single KO-mice had revealed partially conflicting results in regard to atherosclerosis and insulin resistance [3, 9, 13, 16, 17, 29, 32]. In addition, the use of single *Nod*-KO mice potentially harbors the risk of compensatory effects by the other NOD member. To address the impact of *Nod1* and *Nod2* on experimental atherosclerosis and lipid metabolism, we crossed *Nod1*- and *Nod2*-deficient mice on the background of *Ldlr*-deficient mice. These mice are abbreviated as *Ldlr*^−/−^*Nod1/2*^−/−^ in the following; *Ldlr*^−/−^ mice served as control. To induce hypercholesterolemic conditions, mice were fed an HFD for 12 weeks. Body weight gain at the end of the feeding period was slightly more pronounced in *Ldlr*^−/−^*Nod1/2*^−/−^ mice, but did not reach statistical significance (Fig. 1b). Fasting plasma glucose (Fig. 1c) and insulin levels (Fig. 1d) were similar between the groups and we likewise did not observe any effects on glucose tolerance (Fig. 1e) or insulin resistance (Fig. 1f) as possible signs of prediabetes in *Ldlr*^−/−^*Nod1/2*^−/−^ mice. Next, we analyzed plasma lipid levels as a critical parameter in the development of atherosclerosis. Plasma total cholesterol was significantly decreased in *Ldlr*^−/−^*Nod1/2*^−/−^ mice. Subsequent sequential ultracentrifugation analysis revealed that *Ldlr*^−/−^*Nod1/2*^−/−^ mice had significantly lower pro-atherogenic very-low-density lipoprotein (VLDL) cholesterol levels, whereas low-density lipoprotein (LDL), high-density lipoprotein (HDL) and triglyceride (TG) levels were unchanged (Fig. 2a, Online Table 1). To explore the potential underlying mechanism, we next analyzed the hepatic expression levels of key factors for cholesterol and triglyceride metabolism and detected significantly enhanced hepatic expression of scavenger receptor low-density lipoprotein receptor-related protein 1 (*Lrp1*), apolipoprotein A1 (*Apoa1*), apolipoprotein B (*Apob*) and metabolic nuclear receptor liver X receptor alpha (*Lxrα*) in *Ldlr*^−/−^*Nod1/2*^−/−^ mice (Fig. 2b). However, intrahepatic cholesterol level itself was not altered in *Ldlr*^−/−^*Nod1/2*^−/−^ mice (Fig. 2c). In accordance with the results from *Nod1/2*-deficient mice, we found reduced hepatic mRNA expression levels of *Lrp1* and *Lxrα* after injection of the NOD ligands Tri-DAP and MDP into *Nod1/2*-competent mice, whereas *Apoa1* and *Apob* were not changed (Online Fig. 1).

### *Nod1/2-*deficiency elevates intestinal cholesterol levels and alters intestinal microbiota composition

To identify potential mechanisms of an altered cholesterol metabolism in *Ldlr*^−/−^*Nod1/2*^−/−^ mice, we additionally investigated feces samples from the cecum after 12 weeks of HFD. *Ldlr*^−/−^*Nod1/2*^−/−^ mice showed a profound increase in cholesterol and its bacterial degradation products dihydrocholesterol and coprostanol (Fig. 2d). Primary and secondary bile acids were analyzed as well, but did not show any significant differences (Online Table 2).

**Fig. 3:**
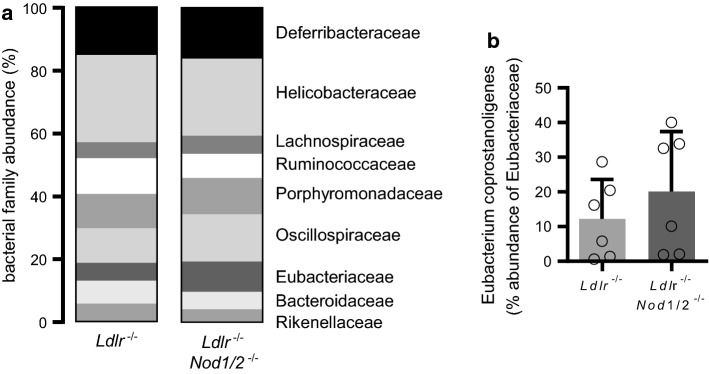
Nod1/2-deficiency alters microbiota composition. *Ldlr*^−/−^ and *Ldlr*^−/−^*Nod1/2*^−/−^ mice were fed a HFD diet for 12 weeks. Feces samples from the cecum were subjected to DNA extraction, purification and barcode labeling. 3rd generation sequencing was performed using Oxford Nanopore Technologies MinION. **a** Bacterial family abundance, *n* = 8. **b** Eubacterium coprostanoligenes as % abundance of Eubacteriaceae. Data were analyzed by Student *t*-test or Mann Whitney test

Next, we investigated if *Nod1/2*-deficiency had an influence on gut microbiota composition under hypercholesteremic conditions. To this end, we used again feces samples from the cecum after 12 weeks of diet. Third-generation sequencing revealed largely consistent gut microbiota composition at the level of bacterial families between the groups (Fig. 3a). However, *Eubacteriaceae* abundance were nearly doubled in *Ldlr*^−/−^*Nod1/2*^−/−^ mice (9.3% vs. 5.4%, Fig. 3a). Within the family of *Eubacteriaceae*, *Eubacterium coprostanoligenes*, which is known to metabolize cholesterol to coprostanol, showed an elevated abundance in *Ldlr*^−/−^*Nod1/2*^−/−^ mice compared to *Ldlr*^−/−^ mice (Fig. 3b).

### *Nod1/2*-deficient mice have reduced atherosclerotic plaque burden and plaque macrophage content

**Fig. 4:**
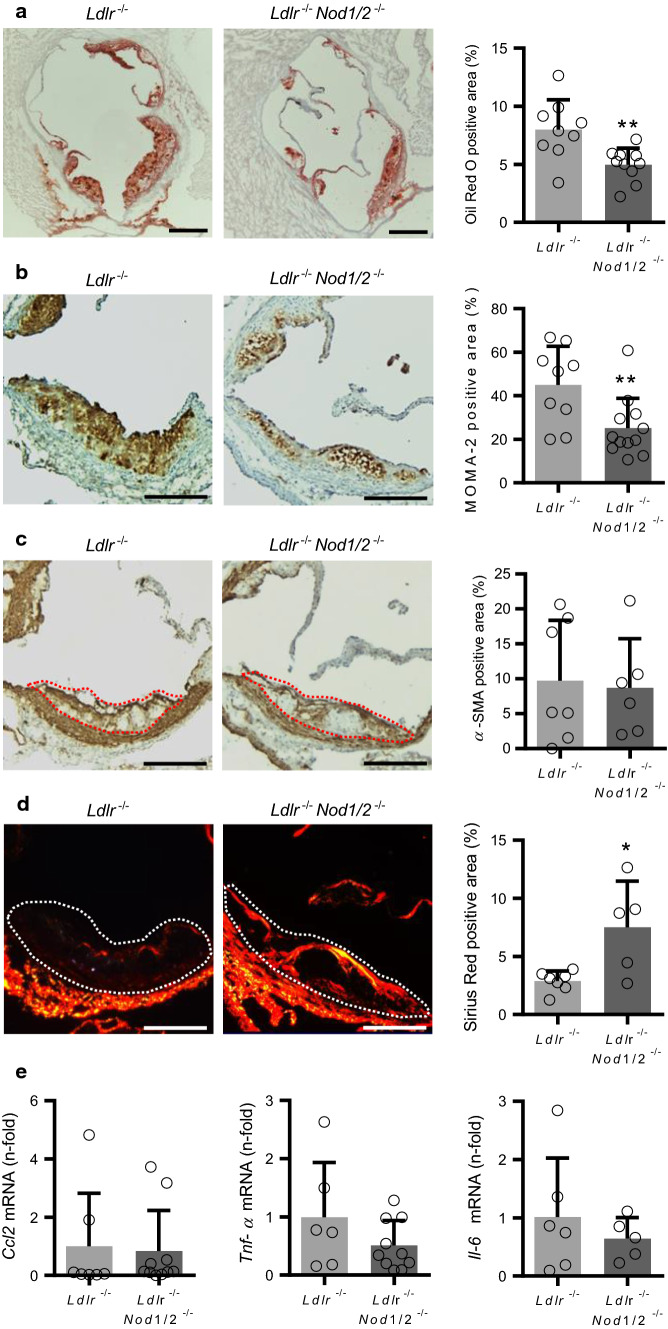
Nod1/2-deficient mice have reduced atherosclerotic plaque burden and plaque macrophage content. *Ldlr*^−/−^ and *Ldlr*^−/−^*Nod1/2*^−/−^ mice were fed a HFD diet for 12 weeks. Sections of aortic roots stained with **a** Oil Red O, antibodies specific for **b** MOMA-2, **c** α-SMA and **d** Sirius Red staining for collagen content in polarized light. Representative pictures are shown, as well as the respective quantification. Oil Red O positive area is expressed as percentage of total surface area of the aortic root and MOMA-2, α-SMA and Sirius Red positive area as percentage of total plaque area. Positive staining for α-SMA and Sirius Red within the vascular wall is not included in the analysis. Plaque area is indicated by a dashed line. Scale bars = 200 µm. **e** *Ccl2*, *Tnf-α* and *Il-6* mRNA levels in aortic arch tissue mice were analyzed by real-time PCR. Data were analyzed by Student *t*-test or Mann Whitney test, *P < 0.05, **P < 0.01

In the *Ldlr*-deficient mouse model, we expected increased vascular inflammation under HFD conditions compared to chow diet (CD), which we exemplified by increased *Tnf-α* expression levels in atherosclerotic aortic tissue. Expression levels of *Nod1* and *Nod2*—likewise involved in inflammation—were similar between CD and HFD conditions (Online Fig. 2a). In the liver, expression levels only for *Nod1* were similar, whereas *Nod2* was significantly enhanced by HFD (Online Fig. 2b). Next we analyzed the atherosclerotic plaque burden after 12 weeks of HFD. Oil Red O staining revealed significantly attenuated lipid deposition in plaques of the aortic root in *Ldlr*^−/−^*Nod1/2*^−/−^ mice compared to *Ldlr*^−/−^ mice (Fig. 4a). However, no differences in lipid deposition in the thoracoabdominal aorta were found (Online Fig. 3). Focusing on the aortic root, we investigated the plaque macrophage content, since infiltrating monocytes and their subsequent differentiation to macrophages represent a crucial step for atherogenesis [15]. Staining for the general monocyte/macrophage marker MOMA-2 revealed reduced macrophage accumulation in plaques of *Ldlr*^−/−^*Nod1/2*^−/−^ mice (Fig. 4b). However, we did not observe any differences in total blood leukocytes between *Ldlr*^−/−^ and *Ldlr*^−/−^*Nod1/2*^−/−^ mice (42,700 ± 11,300 counts vs. 45,700 ± 15,600 counts) or total monocytes and their subsets in peripheral blood and bone marrow (Online Fig. 4). Smooth muscle cell content as an indicator of plaque stability [15] was not affected by *Nod1/2*-deficiency (Fig. 4c). In addition, smooth muscle cells are a major source of stabilizing collagens within atherosclerotic plaques [15]. Therefore, we assessed Sirius Red staining by polarized light microscopy, which displays structural mature type I collagen in bright orange/red and found increased plaque collagen deposition in *Ldlr*^−/−^*Nod1/2*^−/−^ mice (Fig. 4d). In addition, we investigated pro-inflammatory gene expression in aortic arch tissue. We detected similar mRNA levels for C–C motif chemokine ligand 2 (*Ccl2*) but a trend towards decreased levels of *Tnf*-α and *Il-6* in *Ldlr*^−/−^*Nod1/2*^−/−^ mice (Fig. 4e).

### *Nod1/2*-deficiency suppressed oxLDL uptake by macrophages

**Fig. 5:**
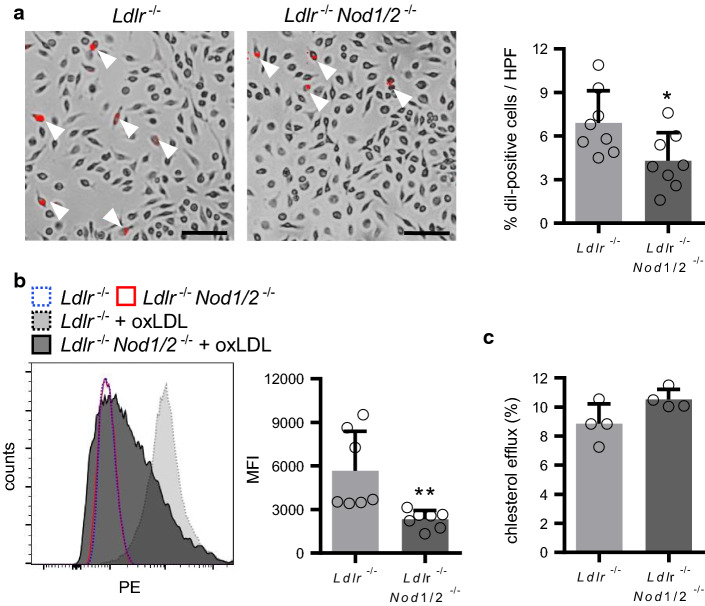
Nod1/2-deficiency suppressed oxLDL uptake by macrophages. Foam cell formation of BMDM from *Ldlr*^−/−^ and *Ldlr*^−/−^*Nod1/2*^−/−^ mice is demonstrated by the uptake of Dil-labelled oxLDL quantified using **a** fluorescence microscopy and **b** flow cytometry. Dil-oxLDL-positive cells are indicated by white arrowheads. Scale bar = 100 µm. Representative pictures are shown. **c** Cholesterol efflux from BMDM was assessed by liquid scintillation counting. Data were analyzed by Student *t*-test or Mann Whitney test, *P < 0.05, **P < 0.01

Since *Ldlr*^−/−^*Nod1/2*^−/−^ mice showed decreased aortic root plaque lipid deposition, we investigated the effect of *Nod1/2*-deficiency on foam cell formation. Uptake of Dil-labeled oxLDL was significantly lower in bone marrow-derived macrophages (BMDM) isolated from *Ldlr*^−/−^*Nod1/2*^−/−^ mice compared to *Ldlr*^−/−^ mice as assessed by microscopy (Fig. 5a) as well as by flow cytometry (Fig. 5b). Real-time PCR analysis in BMDM revealed similar mRNA levels of the LDL uptake receptors macrophage scavenger receptor 1 (*Msr1*), lectin-like oxidized LDL receptor-1 (*Lox-1*) and cluster of differentiation 36 (*Cd36*) between the groups (Online Fig. 5). However, mRNA expression of the LDL efflux transporters ATP-binding cassette transporter A1 (*Abca1*) and G1 (*Abcg1*) were significantly increased in BMDM from *Ldlr*^−/−^*Nod1/2*^−/−^ mice (Online Fig. 5a). Moreover, we observed a trend towards increased cholesterol efflux in BMDM from *Ldlr*^−/−^*Nod1/2*^−/−^ mice (Fig. 5c). We likewise observed a trend towards elevated *Abca1* and *Abcg1* expression levels in the aortic arch, a tissue with a high proportion of plaque macrophages (Online Fig. 5b).

## Discussion

High cholesterol levels and comorbidities such as arterial hypertension or type 2 diabetes are pharmacologically targeted for the treatment of atherosclerosis since a while [5, 15, 26], whereas the inflammatory nature of the disease was targeted for the first time in 2017 [27]. NOD1 and NOD2 are soluble cytosolic immune receptors responsible for the recognition of bacterial peptidoglycan fragments. They share similarities in regard to structure and induction of inflammatory pathways—such as NF-κB and MAPK—in order to initiate inflammatory cytokine expression for immune defense [8, 12, 24, 25]. Based on these similarities, we generated *Nod1*/*Nod2*-double KO mice on a *Ldlr*-deficient background (= *Ldlr*^−/−^*Nod1/2*^−/−^) which showed less lipid deposition and inflammatory cell infiltration in atherosclerotic plaques.

Considering the crucial role of NOD receptors in the initiation of inflammatory pathways, we expected especially an influence of *Nod1/2*-deficiency on vascular inflammation. However, we did not detect significant changes in *Ccl-2*, *Tnf-α* und *Il-6* expression in the aortic arch in both groups. Reduction in plaque burden and monocyte content may be mediated by a lipid phenotype in the current study since *Ldlr*^−/−^*Nod1/2*^−/−^ mice showed reduced cholesterol plasma levels and diminished macrophage foam cell formation. Increased expression of hepatic cholesterol uptake pathways and elevated intestinal cholesterol levels suggested an accelerated cholesterol clearance from the circulation in *Ldlr*^−/−^*Nod1/2*^−/−^ mice. It fits in the picture that the intestinal abundance of the cholesterol-metabolizing *Eubacterium coprostanoligenes*, probably due to more favorable growth conditions, and their metabolic product coprostanol were found to be likewise enhanced. Effects of gut microbiota composition have been already associated with cardiovascular disease including atherosclerosis; however, little is known about the exact mechanisms [2]. An impact of NOD1 and NOD2 on the intestinal microbiota composition [21] also under dietary intake [7] has been already established.

Interestingly, many PRRs initially identified for pathogen recognition and subsequent inflammatory immune response turned out to play crucial roles in pathophysiological conditions including atherosclerosis. First observations were made for Toll-like receptors (TLRs), genetic deletion of *Tlr4* was shown to reduce experimental atherosclerosis in *Apoe*-deficient mice [20]. Later on, similar results have been shown for many other TLRs in related models [18] and likewise for NOD receptors [13, 16, 17, 32] which we confirm in our study using for the first time a *Nod1/2*-double KO model. However, an essential impact of bacterial components itself on atherosclerosis can be ruled out, since none of the conducted large clinical trials with antibiotic treatment in patients with cardiovascular disease demonstrated any long-term benefit [6]. In addition, adverse effects of antibiotic treatment on microbiota composition are conceivable. Later identified endogenous ligands for PRRs, such as degradation products of extracellular matrix components released during tissue damage or apoptotic cell material, rather than live pathogens seem to be responsible for PRR-dependent effects in atherosclerosis. Different to NOD receptors, endogenous ligands for almost every member of the TLR family have been described [14]. Moreover, dietary unsaturated fatty acids such as lauric acid (dodecanoic acid) from coconut oil have been shown to activate TLR4 [31] as well as NOD1 and NOD2 [34], demonstrating a potential influence of PRRs on the development of chronic inflammatory diseases by sensing dietary ingredients.

In the current study, we detected an effect of *Nod1/2*-deficiency on experimental atherosclerosis and lipid metabolism, whereas we did not observe effects on insulin resistance. As already mentioned, the current literature on NOD1 and NOD2 in this field shows partially divergent results [3, 9, 13, 16, 17, 29, 32], which are most likely attributable to different experimental settings such as the animal model used (*Apoe*-KO, *Ldlr*-KO), diet ingredients, feeding period, antibiotic treatment or the use of a specific pathogen challenge.

In addition, some limitations of our study are also worth mentioning. First of all, every murine animal model of experimental atherosclerosis only insufficiently reflects the situation in patients with atherosclerosis. Genetic deletion of certain factors, in our case systemic lifetime deficiency for *Nod1* and *Nod2*, does not represent the physiological situation. We have selected *Ldlr*-deficient mice for our experimental study since their plasma lipoprotein profile, contrary to *Apoe*-deficient mice, more closely resembles that of humans [33]. However, due to deletion of vascular cholesterol clearing pathways, atherosclerosis development is experimentally accelerated and condensed to some weeks, whereas the disease itself develops over decades in humans. Moreover, patients usually die due to rupture of end-stage coronary plaque, which are not existing in mice. Therefore, care must be taken to translate results from experimental mouse studies on atherosclerosis to patients. However, a genome-wide association study has already shown that a rare variant of NOD1 is associated with intima-media thickness in patients [11] suggesting a potential role for the NOD pathway in cardiovascular disease.

Reduced plaque burden in *Ldlr*^−/−^*Nod1/2*^−/−^ mice in our study was restricted to the aortic arch, whereas we did not detect differences in the whole thoracoabdominal aorta, potentially due to different hemodynamic conditions in these distinct vascular beds. This might be also attributed to the use of a HFD containing rather low cholesterol levels (research diets, D12079B), which led on the one hand to moderate plaque load but on the other hand allowed us to simultaneously study metabolic effects.

In summary, we identified NOD1 and NOD2 as pro-atherosclerotic factors, mainly due to their influence on plasma cholesterol levels. Combined deficiency of *Nod1* and *Nod2* reduced lipid-loaded plaque area and plaque macrophage content. In our view, this is conceivably explained by reduced levels of total cholesterol, especially VLDL-cholesterol (the observed effects are depicted in a cartoon in Online Fig. 6). Combined, our results establish NOD1 and NOD2 as another example for evolutionary conserved immune receptors with crucial functions for cholesterol metabolism translating to vascular pathology.

## Electronic supplementary material

Below is the link to the electronic supplementary material.

Supplementary file1 (DOCX 721 kb)
