# Supplementary material for: Deficiency of Nucleotide-binding oligomerization domain-containing proteins (NOD) 1 and 2 reduces atherosclerosis
Source: Basic Res Cardiol. 2020 Jun 25;115(4):47. doi: 10.1007/s00395-020-0806-2 (PMC7316681; doi:10.1007/s00395-020-0806-2)
Supplement: Supplementary file 1 — Supplementary file1 (DOCX 721 kb) [file 395_2020_806_MOESM1_ESM.docx]

**Supplementary material**

**Deficiency of Nucleotide-binding oligomerization domain-containing proteins (NOD) 1 and 2 reduces atherosclerosis**

Ann-Kathrin Vlacil, Jutta Schuett, Volker Ruppert, Muhidien Soufi, Raghav Oberoi, Kinan Shahin, Christian Wächter, Thomas Tschernig, Yu Lei, Fan Liu, Uwe J.F. Tietge, Bernhard Schieffer, Harald Schuett and Karsten Grote

**Supplementary Methods**

**Histochemistry**

Atherosclerotic burden was quantiﬁed in the thoracoabdominal aorta by Oil Red O (Sigma-Aldrich) staining. The formalin-ﬁxed thoracoabdominal aorta was stained with 0.6% propylenglycol-dissolved Oil Red O (2 h at 37°C) and then washed with 85% propylenglycol to remove excess dye. The remaining fat was removed under a stereomicroscope (Stemi DV4, Carl Zeiss Microimaging, Jena, Germany). Subsequently, the aorta was opened longitudinally, pinned on a black silicone-covered dish and photographed under PBS immersion using a stand-equipped camera (EOS 600D, Canon, Tokyo, Japan). The atherosclerotic plaque size was determined by calculating the percentage of the Oil Red O positive area of the thoracoabdominal aorta.

**Flow Cytometry**

To investigate relative abundance of monocyte and monocyte subsets in peripheral blood mononuclear cells and bone marrow, blood was taken retroorbitally from mice and bone marrow cells were isolated from tibia and femur as stated in the method section of the manuscript. Subsequently, cells were stained using PE-labelled CD3e, CD199, NK1.1, CD49b, CD117 (eBioscience, San Diego, CA) and Ly6G (BD Biosciences) as negative selection markers and the monocyte markers CD11b (Alexa Fluor 488-labelled) and Ly6C (PerCP-labelled) (both from eBioscience) to evaluate total monocytes as well as monocyte subsets using flow cytometry. After 30 minutes staining on ice, cells were washed with PBS and 2% FCS and resuspended in 150 µL PBS and 2% FCS. 50 µL of CountBright absolute counting Beads (Thermo Fisher Scientific) were added resulting in a final volume of 200 µL. Dead cells were excluded using DAPI.

Cells were acquired on a FACS LSR II flow cytometer (BD Biosciences, San Jose, CA) and analyzed using Flowjo (Tree Star Inc., Ashland, OR).

**Primer sequences**

Primer sequences used for real-time PCR with SYBR Green PCR Master mixture:

m-*Abca1*-F: 5´-CGTGTCTTGTCTGAAAAAGGAGG-3´

m-*Abca1*-R: 5´-CGTGTCACTTTCATGGTCGC-3´

m-*Abcg1*-F: 5´-GGACTCGGTCCTGACACATC-3´

m-*Abcg1*-R: 5´-CAGGTACAGCAGGCCAATGA-3´

m-*Abcg5*-F: 5´-CTGCATGTGTCCTACAGCGTCA-3´

m-*Abcg5*-R: 5´-AGATGCACATAATCTGGCCACTCTC-3´

m-*Abcg8*-F: 5´-TCAGTCCAACACTCTGGAGGTCA-3´

m-*Abcg*8-R: 5´-ATTTCGGATGCCCAGCTCAC-3´

m-*Apoa1*-F: 5´-GCACGTATGGCAGCAAGATG-3´

m-*Apoa1*-R: 5´-GATTCAGGTTCAGCTGTTGGC-3´

m-*Apob*-F: 5´-TACTTCCACCCACAGTCCCCT-3´

m-*Apob*-R: 5´-CCTTAGAAGCCTTGGGCACAT-3´

m-*Apoc3*-F: 5´-CCATCTAGCCCACAGAAGGC-3´

m-*Apoc3*-R: 5´-TACCTCTTCAGCTCGGGCA-3´

m-*Apoe*-F: 5´-TTTTCCCTCCGCAGACTGG-3´

m-*Apoe*-R: 5´-CTAGGCATCCTGTCAGCAATGT-3´

m-*Cd36*-F: 5´-ATTTGCAACTGAGTGGACTT-3´

m-*Cd36*-R: 5´-GCCAACATGGAACAAATAG-3´

m-*Gapdh*-F: 5´- GTCTCCTGCGACTTCAGC-3´

m-*Gapdh*-R: 5´- TCATTGTCATACCAGGAAATGAGC-3´

m-*Il-1β*-F: 5´-GCCACCTTTTGACAGTGATGAG-3´

m-*Il-1β*-R: 5´-GACAGCCCAGGTCAAAGGTT-3´

m-*Lox-1*-F: 5´-ACCTGCACTCCTTCTTCCCCTTTGT-3´

m-*Lox-1*-R: 5´-GCCTTTGAGCCCTCTGCCTGC-3´

m-*Lpl*-F: 5´-TCGTCATCGAGAGGATCCGA-3´

m-*Lpl*-R: 5´-TGTTTGTCCAGTGTCAGCCA-3´

m-*Lrp1*-F: 5´-ACTTCTCGGATGCCACCTTG-3´

m-*Lrp1*-R: 5´-GGATCACATAGCGGTGGGAG-3´

m-*Lxrα*-F: 5´-GCAGGGAGGACCAGATCGCCTTG-3´

m-Lxrα-R: 5´-CAGCTTCATTAGCATCCGTGGGAAC-3´

m-*Msr1*-F: 5´-CCGACCTTATAGACACGGGACGC-3´

m-*Msr1*-R: 5´-TCCCATGTTCCTGGACTGACGA-3´

m-*Mttp*-F: 5´-TCAGATGGACGCCAGCTTTT-3´

m-*Mttp*-R: 5´-GCCAGTTGTGTGACCGCTAT-3´

m-*Nod1*-F: 5´-GTGGGCTCACCTGTTTGCTA-3´

m-*Nod1*-R: 5´-CTGGTTGACGCTGAGTCTGAT-3´

m-*Nod2*-F: 5´-TGTGGAGTCACCGCAAAACT-3´

m-*Nod2*-R: 5´-GCCTGATCTCCTCACACTCG-3´

m-*Sort1*-F: 5´-ACTTCACTGGGCTTGCTTCC-3´

m-*Sort1*-R: 5´-CCTCTTCACAATTCCGCTCA-3´

m-*Sr-b1*-F: 5´-TTGGCCTGTTTGTTGGGATG-3´

m-*Sr-b1*-R: 5´-GGATTCGGGTGTCATGAAGG-3´

m-*Tnf-α*-F: 5´- CTGGCACCACTAGTTGGTTGT -3´

m-*Tnf-α*-R: 5´- GTAGCCCACGTCGTAGCAAAC-3´

Primer and probe sequences used for real-time PCR with TaqMan Fast Advanced MasterMix:

m-*Ccl2*-F: 5´-CTCAGCCAGATGCAGTTAA-3´

m-*Ccl2*-R: 5´-CTCTCTTGAGCTTGGTGAC-3´

m-*Ccl2*-P: 5´-TCTTTGGGACACCTGCTGCT-3´

m-*Gapdh*-F: 5´-GGAGAAACCTGCCAAGTATG-3´

m-*Gapdh*-R: 5´-CCTCAGTGTAGCCCAAGA-3´

m-*Gapdh*-P: 5´-AGATGCCTGCTTCACCACCTTCTTG-3´

m-*Il-6*-F: 5´-CGCTATGAAGTTCCTCTCTG-3´

m-*Il-6*-R: 5´-GCCATTGCACAACTCTTTTC-3´

m-*Il-6*-P: 5´-TCCATCCAGTTGCCTTCTTGGGACT-3´

m-*Tnf-α*-F: 5´-GTCTCAGCCTCTTCTCATTC-3´

m-*Tnf-α*-R: 5´-CTTGGTGGTTTGCTACGA-3´

m-*Tnf-α*-P: 5´-CCACCACGCTCTTCTGTCTACTGAA-3´

**Online Fig. 1** Analysis of NOD-stimulated hepatic gene expression of selected key factors for cholesterol metabolism. *Ldlr^−/−^* mice were i.v. injected with the NOD1 agonist Tri-DAP or the NOD2 agonist MDP (100 µg/mouse) and hepatic *Lrp1*, *Lxrα*, *Apoa1* and *Apob* mRNA levels were determined by real-time PCR after 6 hrs. **P*<0.05, **P<0.01 vs. control (con). Data were analyzed by Student t-test or Mann Whitney test.

**Online Fig. 2** Analysis of *Tnf-α, Nod1* and *Nod2* expression. *Ldlr^−/−^* mice were fed a chow diet (CD) or a high fat diet (HFD) for 12 weeks. *Tnf-α, Nod1* and *Nod2* mRNA levels in **a** aortic arch tissue and *Nod1* and *Nod2* mRNA levels in **b** the liver were determined by real-time PCR. **P<0.01. Data were analyzed by Student t-test or Mann Whitney test.

**Online Fig. 3** Analysis of atherosclerotic plaque burden in the thoracoabdominal aorta. *Ldlr*^−/−^ and *Ldlr^−/−^Nod1/2^−/−^* mice were fed a high fat, high cholesterol diet for 12 weeks. En face preparations of thoracoabdominal aortas stained with Oil Red O. Representative pictures are shown, as well as the respective quantiﬁcation. Oil Red O positive area is expressed as percentage of total surface area thoracoabdominal aorta. Scale bars=2 mm. Data were analyzed by Student t-test or Mann Whitney test.

**Online Fig. 4** Monocytes in peripheral blood and bone marrow. **a** Flow cytometry gating strategy for bone marrow monocyte subsets according to Ly6C surface expression. Cells negative for CD3e, CD19, CD49b, CD117, Ly6G and NK1.1 and positive for CD11b were selected. The counts of total CD11b^+^ monocytes and their subsets according to Ly6C surface expression in **b** peripheral blood and **c** bone marrow of *Ldlr*^−/−^ and *Ldlr^−/−^Nod1/2^−/−^* mice was quantified by flow cytometry. FSC = forward scatter, SSC = side scatter. Data were analyzed by Student t-test or Mann Whitney test.

**Online Fig. 5** LDL receptor and efflux transporter expression in BMDM and aortic arch tissue. *Ldlr^−/−^ and Ldlr^−/−^Nod1/2^−/−^* were fed a HFD for 12 weeks. *Msr1*, *Lox-1*, *Cd36*, *Abca1* and *Abcg1* mRNA levels in **a** BMDM and *Abca1* and *Abcg1* mRNA levels in **b** aortic arch tissue were determined by real-time PCR. *P<0.05. Data were analyzed by Student t-test or Mann Whitney test.

**Online Fig. 6** Effects of Nod1/2-deficiency on atherosclerosis and cholesterol metabolism under hypercholesterolemic conditions.

**Table 1.** Plasma lipid levels after 12 weeks of HFD.

*Ldlr^−/−^*  *Ldlr^−/−^Nod1/2^−/−^*  p-value

triglycerides 262±51 270±43 n.s.

total cholesterol 1155±102 844±142 <0.01

VLDL-C 611±68 389±77 <0.05

LDL-C 439±30 360±53 n.s.

HDL-C 106±7 95±12 n.s.

Plasma lipid levels are given in mg/dL. VLDL-C=very-low-density lipoprotein cholesterol, LDL-C=low-density lipoprotein cholesterol, HDL-C=high-density lipoprotein cholesterol. N=8-9, n.s.=not significant.

**Table 2.** Primary and secondary bile acids in feces samples from the cecum after 12 weeks of HFD.

*Ldlr^−/−^*  *Ldlr^−/−^Nod1/2^−/−^*  p-value

α-muricholic acid 2.74±0.67 3.17±0.53 n.s.

deoxycholic acid 2.50±0.59 3.60±0.64 n.s.

cholic acid 4.53±1.60 3.15±0.56 n.s.

chenodeoxycholic acid 0.23±0.10 0.18±0.05 n.s.

hyodeoxycholic acid 0.17±0.03 0.26±0.06 n.s.

ursodeoxycholic acid 0.20±0.07 0.21±0.05 n.s.

β-muricholic acid 4.37±1.29 3.43±0.85 n.s.

ω-muricholic acid 2.89±0.44 4.09±0.40 n.s.

N=8-14, n.s.=not significant.
